# Medical Gases as Emerging Regulators of Paediatric Endocrine and Neurodevelopmental Pathways: A Mini‐Review

**DOI:** 10.1111/apa.70429

**Published:** 2026-01-03

**Authors:** Roberto Paparella, Fabiola Panvino, Ida Pucarelli, Luigi Tarani

**Affiliations:** ^1^ Department of Maternal Infantile and Urological Sciences Sapienza University of Rome Rome Italy; ^2^ Department of Human Neuroscience, Section of Child and Adolescent Neuropsychiatry Sapienza University of Rome Rome Italy

**Keywords:** gasotransmitters, medical gases, neurodevelopment, oxidative stress, paediatric endocrinology

## Abstract

**Aim:**

Medical gases, including nitric oxide, carbon monoxide, hydrogen sulphide and molecular hydrogen, have emerged as key regulators of redox balance and cellular signalling. This mini‐review examines their relevance to paediatric endocrine and neurodevelopmental pathways, domains particularly sensitive to oxidative and inflammatory disturbances.

**Methods:**

We surveyed preclinical and clinical studies published between 2007 and 2025 on gas‐mediated regulation of metabolic–redox homeostasis, bone biology, pubertal control and neurodevelopment. Additional attention was given to conditions marked by oxidative stress, such as Klinefelter and Turner syndromes.

**Results:**

Evidence shows that gasotransmitters modulate synaptic plasticity, neurotransmission and neuroinflammation, influencing disorders such as autism spectrum disorder, attention‐deficit/hyperactivity disorder and outcomes after perinatal hypoxia. They also participate in metabolic regulation, osteogenesis, osteoclast activity and hypothalamic control of puberty. These mechanistic insights highlight the emerging translational potential of gas‐mediated pathways in paediatric health.

**Conclusion:**

Although paediatric clinical applications remain limited, advances in omics‐based profiling, mechanistic studies and biomaterial‐supported gas delivery are rapidly expanding the therapeutic horizon. Integrating gasotransmitter biology into paediatric endocrinology and neurodevelopment may support future diagnostic, preventive and targeted therapeutic strategies.

## Summary

Research on medical gases in paediatric populations is needed because their roles in growth, metabolism, endocrine function and neurodevelopment remain poorly understood.

This mini‐review found that nitric oxide, carbon monoxide, hydrogen sulphide and molecular hydrogen regulate redox balance, skeletal and pubertal development and neural plasticity.

These findings suggest that gas‐mediated pathways may support the development of new diagnostic tools and therapeutic strategies for paediatric endocrine and neurodevelopmental disorders.

**Abbreviations**
CO: carbon monoxide
H_2_
: molecular hydrogen
H_2_S: hydrogen sulphide
NO: nitric oxide

## 1 Introduction

Medical gas research has emerged as a dynamic and interdisciplinary field that has extended far beyond its traditional applications in respiratory medicine and anaesthesia. Over the last two decades, gasotransmitters, a term first introduced by Wang in 2002 [1], have included nitric oxide (NO), carbon monoxide (CO), hydrogen sulphide (H_2_S) and molecular hydrogen (H_2_). These gases have been redefined from toxic by‐products to signalling molecules with regulatory functions in human physiology. They influence redox balance, mitochondrial activity, vascular tone and cellular communication and this has opened new avenues in oncology, neurology, regenerative medicine and biomaterials science.

A major turning point in this field occurred when nitric oxide was identified as the endothelium‐derived relaxing factor. Subsequent studies clarified its mechanisms of action and contributed to the awarding of the Nobel Prize in Physiology or Medicine in 1998 to Furchgott, Ignarro and Murad [2, 3, 4].

Despite this progress, the role of medical gases in paediatric endocrinology, metabolism and neurodevelopment remains underexplored. Processes such as bone growth, pubertal development, metabolic regulation and brain maturation are highly dynamic in children and adolescents. These processes are also particularly sensitive to oxidative stress, inflammation and alterations in cellular signalling, and medical gases have been shown to interact with these pathways [5].

There is a strong rationale to expand research on medical gases within paediatric and translational medicine. This applies to rare genetic syndromes, growth and pubertal disorders, metabolic dysregulation and neurodevelopmental conditions [5]. Advances in omics technologies, bioinformatics and large‐scale medical databases have created new opportunities to investigate gas‐mediated mechanisms and identify potential biomarkers in paediatric populations.

## 2 The Mini‐Review

This mini‐review provides an overview of recent advances in the field of medical gases as regulators of paediatric endocrine and neurodevelopmental pathways. We examined preclinical and clinical studies published between 2007 and 2025, excluding historical papers cited for contextual background, such as those introducing the term gasotransmitter or describing the foundational discoveries recognised by the Nobel Prize. The review focuses on evidence that links NO, CO, H_2_ S and H_2_ with redox regulation, growth, metabolism, pubertal development and brain maturation in children. The aim was to synthesise emerging concepts and highlight knowledge gaps relevant to paediatric health and future research.

## 3 Endogenous Gasotransmitters and Metabolic–Redox Homeostasis

One of the key insights of recent years has been the recognition that NO, CO and H_2_S function as gasotransmitters that orchestrate intracellular signalling networks regulating redox balance and mitochondrial activity [6, 7, 8]. These pathways are central to metabolic and endocrine processes in children.

Oxidative stress has been identified as a driver of endocrine and metabolic dysfunction in paediatric populations, including those with sex chromosome aneuploidies such as Klinefelter syndrome and Turner syndrome, as well as children born small for gestational age, and those with obesity or nonalcoholic fatty liver disease [6, 9]. Studies have shown that gasotransmitters modulate antioxidant defences and influence transcription factors like Nrf2 and NF‐κB, which shape cellular resilience to oxidative injury [10, 11, 12]. For example, H_2_S donors restored glutathione levels and promoted mitochondrial biogenesis, while NO displayed concentration‐dependent actions that were protective at physiological levels and harmful when excessive [6, 13].

The gut microbiome represents an additional endogenous source of gasotransmitters. Commensal bacteria produce hydrogen, methane and H_2_S through fermentation and sulphur‐metabolism pathways, which contribute to the systemic gasotransmitter pool [14]. Dysbiosis alters microbial H_2_S production and affects epithelial integrity, mitochondrial respiration, metabolic inflammation and gut–brain communication [14, 15]. Altered microbial gas production in children has been linked to metabolic disturbances, small intestinal bacterial overgrowth, obesity and neurodevelopmental conditions such as autism spectrum disorder. The microbiome–gut–brain axis is therefore an important modulator of circulating gasotransmitters and adds complexity to metabolic–redox homeostasis during growth and development [16].

The clinical relevance of these mechanisms is clear. Children with Klinefelter syndrome show elevated oxidative stress and metabolic vulnerability before puberty, which suggests an opportunity for early interventions aimed at modulating gasotransmitter activity. Small‐for‐gestational‐age infants and adolescents may also experience long‐term metabolic consequences influenced by gas‐regulated vascular and mitochondrial functions [9, 17].

Evidence to date does not support routine clinical use of medical gas interventions such as exogenous NO, CO or H_2_S donors to improve metabolic outcomes in paediatric endocrine disorders. Preclinical and early‐phase clinical studies indicated possible benefits of H_2_S and NO donors in reducing oxidative stress and enhancing mitochondrial function, but these strategies remain investigational and are not established in paediatric practice [18, 19]. Antioxidant therapies, including vitamin C, vitamin E and glutathione precursors, together with syndrome‐specific hormonal treatments, currently represent the most evidence‐based options for restoring redox balance in children with sex chromosome aneuploidies and related metabolic conditions [9].

## 4 Medical Gases in Bone Health, Growth and Puberty

Medical gas research is also relevant to skeletal biology and pubertal development. Paediatric growth depends on endocrine signals such as growth hormone, insulin‐like growth factor 1 and sex steroids, as well as genetic factors and the local microenvironment of the growth plate and bone tissue [20]. Gasotransmitters intersect with several of these regulatory networks.

NO and H_2_S act as potent modulators of osteoblast and osteoclast activity. Experimental studies showed that NO enhanced bone formation through cyclic guanosine monophosphate pathways, while H_2_S promoted osteogenesis through the Wnt/beta‐catenin axis and reduced osteoclast‐mediated resorption [21, 22]. These findings suggested that gasotransmitters may influence bone mineral density, which is clinically relevant in idiopathic short stature, Turner syndrome and Klinefelter syndrome, where osteopenia and fracture risk are well‐recognised concerns.

The pubertal transition, which is a period of rapid skeletal accrual, may also be shaped by gas‐mediated signalling at the hypothalamic, pituitary and gonadal axis. NO plays a direct role in regulating gonadotropin‐releasing hormone secretion and pubertal timing. Neuronal NO synthase activity in the hypothalamus is regulated by oestrogens and is essential for the activation of gonadotropin‐releasing hormone neurons during minipuberty and pubertal onset. NO coordinates the activity and pulsatile release of these neurons, which affects pubertal timing [23, 24, 25]. Disrupted NO signalling caused by genetic, epigenetic or environmental factors can alter gonadotropin‐releasing hormone secretion and may contribute to precocious or delayed puberty, both frequent challenges in paediatric endocrinology [26, 27].

Synergy between medical gases and biomaterials may offer new opportunities for paediatric bone health. Scaffolds that incorporate NO donors or H_2_S‐releasing compounds can support bone regeneration by promoting osteogenesis, angiogenesis and local immune modulation. Hydrogel and nanomaterial delivery systems provide controlled and localised release of gasotransmitters, which may improve bone repair after fractures or surgery and enhance outcomes in paediatric orthopaedic care [28, 29, 30].

## 5 Medical Gases in Neurodevelopment

The nervous system is one of the most gas‐sensitive organs. Its maturation during childhood and adolescence provides an important context for studying the roles of NO, CO and H_2_ in synaptic plasticity, neurotransmission and neuroprotection. These gases modulate calcium signalling, cyclic nucleotides and inflammatory pathways, which positions them as central regulators of neurodevelopmental processes [31].

Gasotransmitters influence synaptic signalling and neuroinflammation in paediatric neurological and psychiatric disorders such as autism spectrum disorder, attention deficit hyperactivity disorder and intellectual disability. They affect long‐term potentiation, oxidative homeostasis and immune responses. NO is a key modulator of long‐term potentiation, which is essential for learning and memory. Excessive NO production and abnormal S‐nitrosylation disrupt glutamatergic transmission in autism spectrum disorder and lead to behavioural and synaptic deficits. Inhibition of neuronal NO synthase can reverse these abnormalities. Physiological NO supports antioxidant defences, whereas excessive NO promotes nitrosative stress and neuroinflammation [32, 33, 34].

H_2_S supports long‐term potentiation by enhancing N‐methyl‐d‐aspartate receptor activity. It also modulates the release of neurotransmitters such as gamma‐aminobutyric acid, glutamate and D‐serine, and protects neurons from oxidative and inflammatory injury. Impaired H_2_S signalling has been linked to autism spectrum disorder and related conditions. Restoring H_2_S levels improves synaptic function and behavioural outcomes in experimental models [35, 36]. H_2_ shows antioxidant and anti‐inflammatory effects that may mitigate neurodevelopmental damage, although evidence remains limited [37].

The interplay between medical gases and neurotrophins, including brain‐derived neurotrophic factor, influences neuronal survival and plasticity in children with genetic syndromes, perinatal hypoxia or traumatic brain injury. Medical gases can enhance neurotrophic signalling through activation of redox‐sensitive transcription factors such as Nrf2. This supports brain‐derived neurotrophic factor expression and promotes neuroplasticity [38, 39].

The safety and feasibility of hydrogen inhalation have been demonstrated in translational studies. Adjunctive use with established therapies such as therapeutic hypothermia is under evaluation. Current evidence supports its potential role in paediatric neuroprotection, particularly in perinatal hypoxia and brain injury, although clinical trials in rare genetic syndromes and cognitive rehabilitation are still limited [40, 41].

CO exhibits a dual role in the developing nervous system. Physiological CO produced through the haeme oxygenase‐1 pathway acts as a signalling molecule. It regulates synaptic plasticity, inflammation and mitochondrial function through cyclic guanosine monophosphate‐dependent and redox‐sensitive pathways [42, 43]. At higher concentrations, CO is a potent neurotoxin, especially in children. It binds strongly to haemoglobin, with an affinity more than 200 times that of oxygen, forming carboxyhaemoglobin and causing tissue hypoxia. CO also binds to cytochrome c oxidase and impairs oxidative phosphorylation, creating excitotoxicity, oxidative stress and delayed neuronal apoptosis. Paediatric CO poisoning is a major cause of preventable brain injury and is associated with acute encephalopathy, persistent cognitive deficits, behavioural disturbances and delayed leukoencephalopathy [44].

The rationale for hyperbaric oxygen therapy illustrates the importance of CO–mitochondria interactions. Hyperbaric oxygen accelerates CO dissociation, improves mitochondrial function and modulates oxidative stress. Despite these insights, controlled studies on regulated CO delivery or haeme oxygenase‐1 modulation in children remain scarce, and its potential therapeutic roles must be interpreted with caution [45].

## 6 Hormesis and Toxicity

The challenges associated with precise and targeted delivery of gasotransmitters in paediatric populations are multifactorial. The risk of off‐target effects is substantial because gases such as NO, CO and H_2_S diffuse rapidly through tissues and cell membranes. This makes it difficult to confine their activity to specific sites and increases the likelihood of unintended toxicity, especially in developing organs [19].

A fundamental concept in medical gas biology is hormesis. All major gasotransmitters display a biphasic dose–response pattern. Physiological concentrations provide cytoprotective, metabolic, or anti‐inflammatory effects. Higher concentrations may induce oxidative injury, inhibit mitochondrial respiration, or disrupt cellular signalling. H_2_S is a clear example. Low doses support mitochondrial function and antioxidant defences, whereas high concentrations inhibit cytochrome c oxidase and act as potent mitochondrial toxins. NO and CO show a similar shift from regulatory mediators to drivers of nitrosative or oxidative stress when present in excess [46].

Maintaining a safe therapeutic window is technically challenging. Small deviations in dose or release kinetics may shift the balance from benefit to harm [47]. These risks are particularly relevant in paediatric populations because organ immaturity and evolving redox systems can increase susceptibility to toxic effects.

Ethical and practical challenges in paediatric clinical trials further complicate the evaluation of medical gases. Children are a vulnerable population and require rigorous consent procedures, age‐appropriate study designs and precise biomarker selection. Small sample sizes, developmental variability and the need for specialised infrastructure limit feasibility and generalisability. As a result, high‐quality safety and efficacy data for gasotransmitter‐based therapies in children remain scarce [48, 49].

## 7 Future Perspectives and Interdisciplinary Proposals

There are several key priorities for future research on medical gases in paediatric medicine.

To begin with, research should clarify the roles of NO, CO, H_2_S and H_2_ in growth, pubertal transition, neurodevelopment, endocrinology and metabolism. This requires mechanistic studies to explain how these gases regulate redox balance, mitochondrial function and cellular signalling in developing tissues, particularly in conditions such as sex chromosome aneuploidies, small‐for‐gestational‐age status, obesity and other metabolic disorders.

Another key priority is to establish safety, efficacy and optimal dosing of medical gases for paediatric use. Most available data were generated in adult or neonatal models. Well‐designed paediatric trials are needed to establish age‐appropriate delivery methods and long‐term outcomes, especially for neuroprotection, metabolic modulation and endocrine support.

A further objective concerns the development of biomaterials tailored to children. Stimuli‐responsive gas‐generating platforms and metal–organic frameworks may permit controlled and site‐specific release of therapeutic gases, which could support bone regeneration, wound healing and targeted treatments.

Finally, efforts are needed to identify reliable biomarkers and diagnostic tools based on gasotransmitter profiles. Noninvasive measurement of exhaled or tissue gases may assist with early diagnosis, risk stratification and monitoring of disease progression.

Interdisciplinary collaboration will be essential to advance this field. Bringing together expertise from paediatrics, molecular biology, genetics, bioinformatics and material sciences will foster robust mechanistic studies, improve patient stratification and support the design of paediatric delivery systems. This approach will also help translate preclinical discoveries into safe and effective therapies for children and adolescents.

## 8 Conclusion

Medical gases have emerged as important regulators of endocrine and neurodevelopmental pathways in children. Evidence shows their contribution to redox balance, metabolic regulation, skeletal development, pubertal timing and neural plasticity. These findings indicate that gas‐mediated mechanisms may play a broader role in paediatric health than previously recognised. Clinical translation remains limited, but advances in mechanistic research, omics technologies and biomaterial‐based delivery systems are creating new opportunities. Continued investigation may support the development of diagnostic tools and targeted therapies for paediatric endocrine and neurodevelopmental disorders.

## Author Contributions

**Fabiola Panvino:** conceptualization, writing – original draft. **Luigi Tarani:** supervision. **Roberto Paparella:** conceptualization, writing – original draft.

## Funding

The authors have nothing to report.

## Conflicts of Interest

The authors declare no conflicts of interest.

## Data Availability

Data sharing not applicable to this article as no datasets were generated or analysed during the current study.
